# Supplementary material for: Observing Dynamic Conformational Changes within the Coiled-Coil Domain of Different Laminin Isoforms Using High-Speed Atomic Force Microscopy
Source: Int J Mol Sci. 2024 Feb 6;25(4):1951. doi: 10.3390/ijms25041951 (PMC10888245; doi:10.3390/ijms25041951)
Supplement: Supplementary file 1 [file ijms-25-01951-s001.zip › Supplementary Material.pdf]

Supplementary Figures

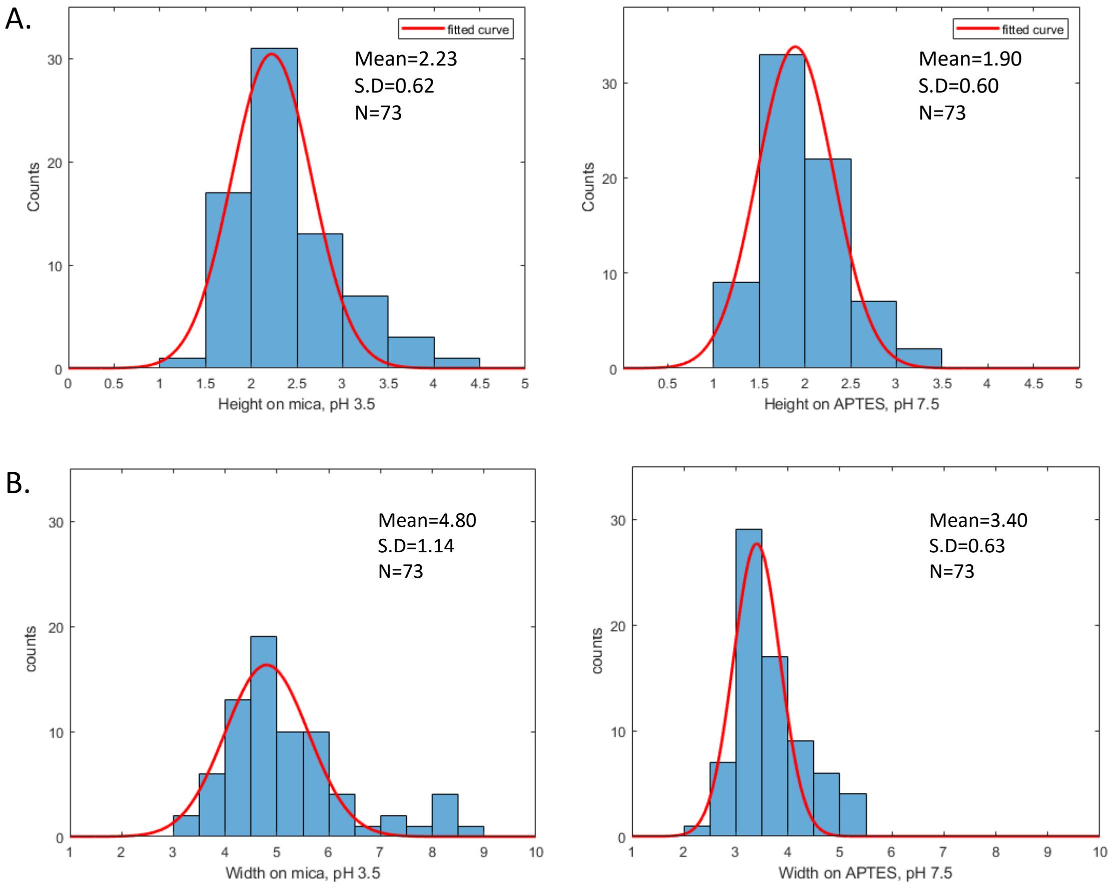

Suppl. Fig S1: Laminin coiled-coil height and width on mica and APTES surfaces. (A) Laminin-111 coiled-coil height distribution on mica imaged at pH 3.5 (left panel) and on APTES-mica at pH 7.5 (right panel). **(B)** Laminin-111 coiled-coil full width at half maximum (FWHM) height distribution on mica at pH 3.5 (right panel) and on APTES-mica at pH 7.5 (left panel). Red lines indicate Gaussian fits to the distributions. N is the number of molecules analyzed for the histogram. S.D. is standard deviation.

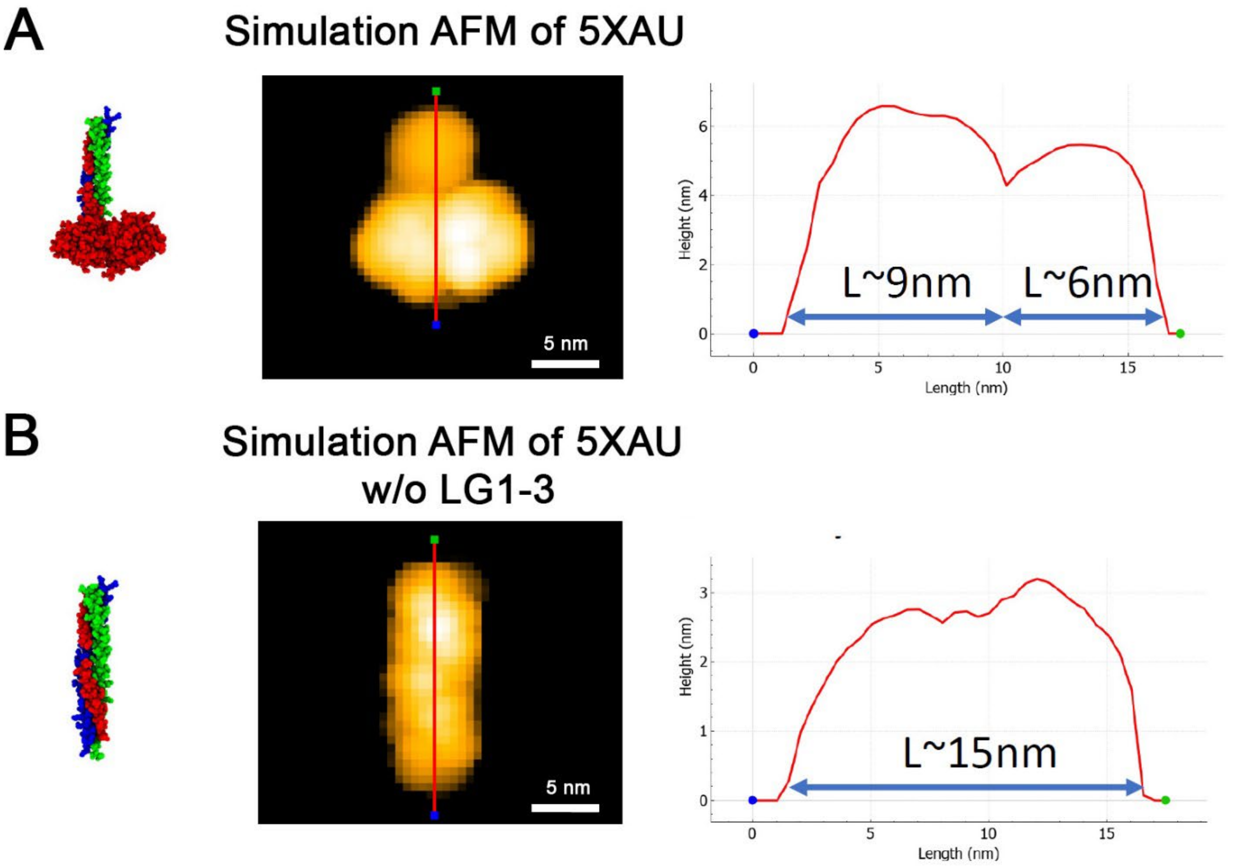

Suppl. Fig S2: Simulation AFM of 5XAU. (A) Crystal structure and simulated AFM image (tip radius = 2 nm) of laminin-511 fragment 5XAU containing the C-terminus of the coiled-coil and the LG1-3 domains. The laminin α5, β1, and γ1 chains are colored in red, blue, and green, respectively. The red line in the Simulation AFM image indicates the position of the height profile line shown on the right. **(B)** The 5XAU structure and corresponding simulation AFM image after removal of LG1-3. The red line in the Simulation AFM image indicates the position of the height profile line shown on the right. Approximately 9 nm of the C-terminal coiled-coil domain are obscured by the overlying LG1-3 domains in AFM topographs.

Supplementary Video legends

Video S1. Laminin-111 at different pH. HS-AFM movies of laminin-111 at pH 3.5, pH 5, pH 6, pH 7.5, pH 9, and pH 10 on bare mica. Images were acquired at 2 sec per frame.

Video S2. Laminin-111 at different APTES concentrations. HS-AFM movies of laminin-111 on mica coated with 1%, 0.1%, and 0.01% APTES. Images acquired at 3 sec per frame.

Video S3. Comparison of laminin-111 on APTES and mica. HS-AFM image sequences of laminin-111 on a bare mica imaged at pH 3.5 (left) and on 0.1% APTES-coated mica imaged at pH 7.5. Images were recorded at 1 sec per frame. Scale bars 40 nm.

Video S4. Observation of Laminin-111 by HS-AFM. HS-AFM movies of three representative laminin-111 molecules on APTES-coated mica imaged at pH 7.5.

Video S5. Observation of Laminin-3A32 by HS-AFM. HS-AFM movies of laminin-3A32 on bare mica and APTES-coated mica imaged at pH 7.5. Images were recorded at 2 sec per frame. Scale bars 100 nm. The green circle denotes a single laminin-3A32 undergoing a jackknife transition between straight and kinked conformations.

Video S6. Observation of Laminin-3B32 by HS-AFM. HS-AFM movies of laminin-3B32 on bare mica and APTES-coated mica imaged at pH 7.5. Images were recorded at 3 sec per frame. Scale bars 150 nm.

Video S7. Visualizing conformation changes in the Laminin-332 coiled-coil domain by HS-AFM. HS-AFM movies of laminin-3A32 (left) and laminin-3B32 (right) molecules undergoing transitions from extended to bent coiled-coil conformations. Scale bars 25 nm.

Video S8. Visualizing laminin-111 LG1-5 domains by HS-AFM. Uncropped (left) and cropped (right) HS-AFM image sequences of laminin-111 displaying conformational fluctuations of the LG1-5 domains. Scale bars 15 nm.

Video S9. Visualizing laminin-3B32 LG1-3 domains by HS-AFM. Uncropped (left) and cropped (right) HS-AFM movies of laminin-3B32 isplaying conformational fluctuations of the LG1-3 domains. Scale bars 20 nm.
