# Supplementary figures and images for: Observing Dynamic Conformational Changes within the Coiled-Coil Domain of Different Laminin Isoforms Using High-Speed Atomic Force Microscopy
Source: Int J Mol Sci. 2024 Feb 6;25(4):1951. doi: 10.3390/ijms25041951 (PMC10888245; doi:10.3390/ijms25041951)

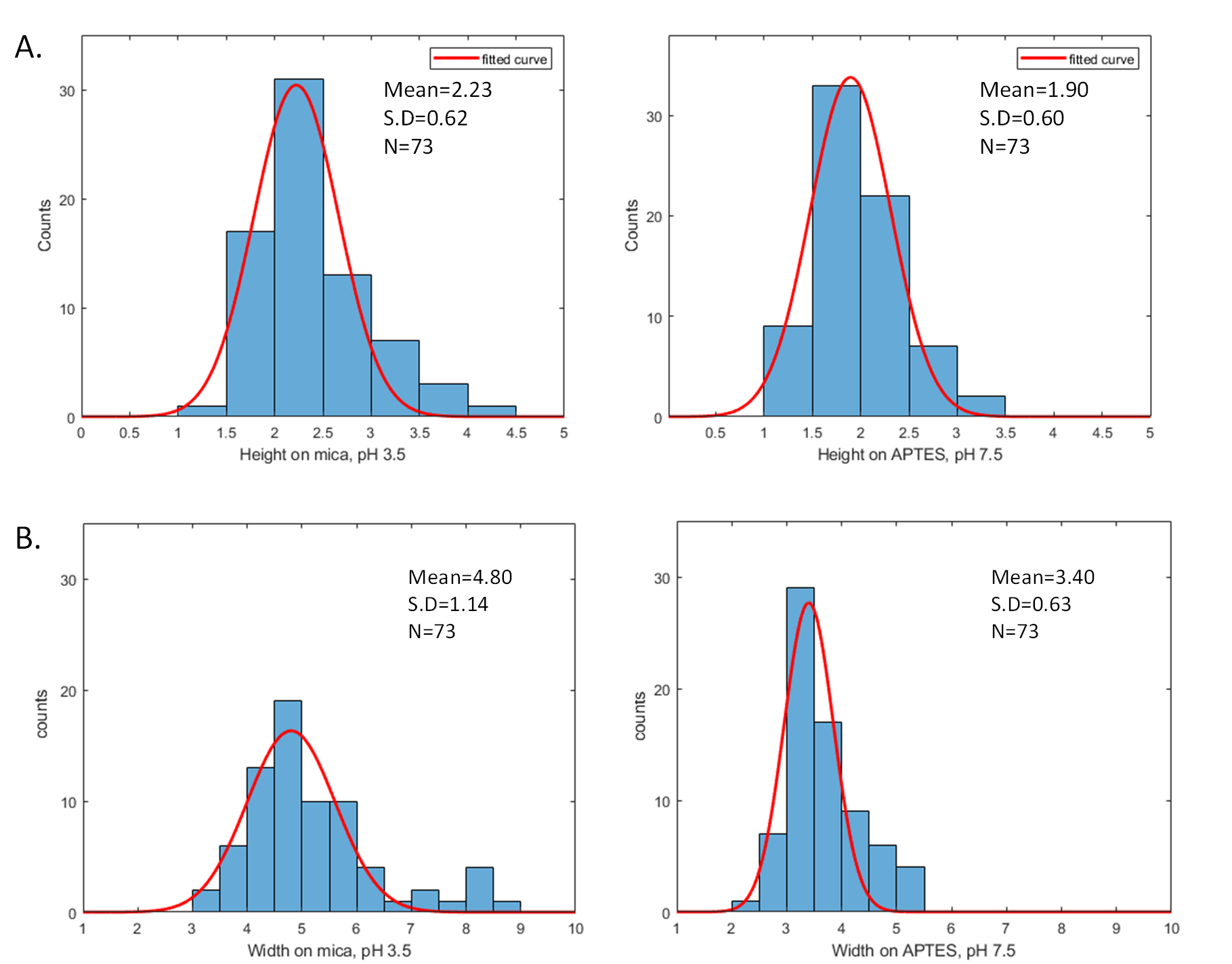

Supplement: Supplementary file 1 [file ijms-25-01951-s001.zip › Figure S1.tif]

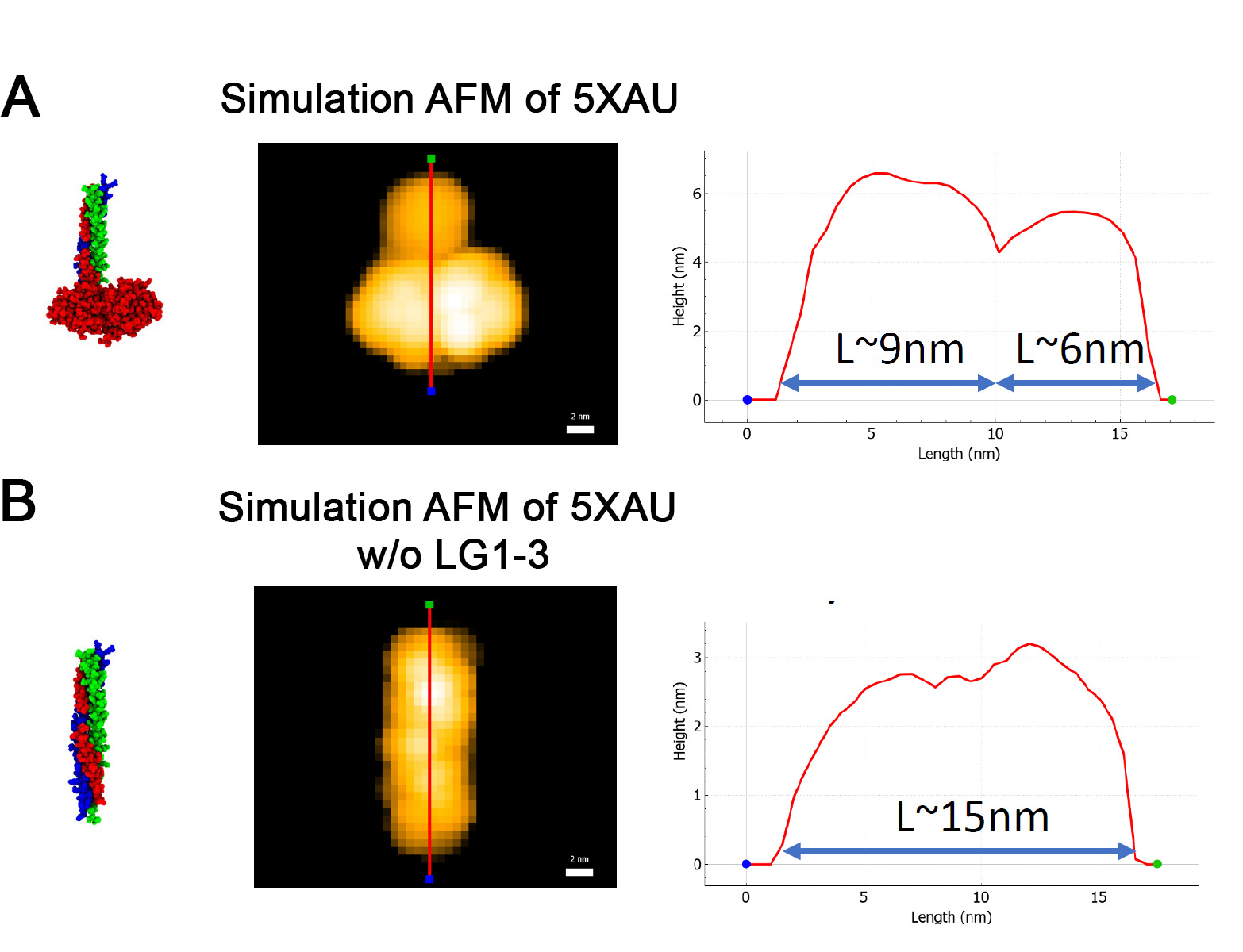

Supplement: Supplementary file 1 [file ijms-25-01951-s001.zip › Figure S2.tif]
